# Integrative Analysis of Methylation and Transcriptional Profiles to Reveal the Genetic Stability of Cashmere Traits in the T*β*4 Overexpression of Cashmere Goats

**DOI:** 10.3390/ani9121002

**Published:** 2019-11-20

**Authors:** Bai Dai, Meng Zhang, Jian-Long Yuan, Li-Qing Ren, Xiao-Yu Han, Dong-Jun Liu

**Affiliations:** State Key Laboratory of Reproductive Regulation and Breeding of Grassland Livestock, School of Life Sciences, Inner Mongolia University, Hohhot 010070, China; 0141124820@mail.imu.edu.cn (B.D.); 21608012@mail.imu.edu.cn (M.Z.); lianghao@imu.edu.cn (J.-L.Y.); 31708012@mail.imu.edu.cn (L.-Q.R.); 0141122082@mail.imu.edu.cn (X.-Y.H.)

**Keywords:** T*β*4, genetic stability, methylation, transcriptome, somatic cell nuclear transfer

## Abstract

**Simple Summary:**

Cashmere goats have double coats consisting of non-medullated fine inner hairs or cashmere fibers produced by secondary hair follicles (SHFs) and guard hairs produced by primary hair follicles (PHFs). Cashmere is an important economic product worldwide and the world market for cashmere is increasing while the current production of cashmere is limited. Thymosin *β*4 (T*β*4), a 4.9-kDa protein, contains 43 amino acids. Here, we produced T*β*4 overexpression (T*β*4-OE) offspring using two methods. The somatic cell nuclear transfer (SCNT) goats had increased hair follicle development and higher cashmere yields than wild type (WT) and natural mating (NM) goats. Taken together, our results showed that DNA methylation affected the expression of differentially expressed genes (DEGs) between generations and the genetic stability of cashmere traits.

**Abstract:**

DNA methylation alteration is frequently observed in exogenous gene silencing and may play important roles in the genetic stability of traits. Cashmere is derived from the secondary hair follicles (SHFs) of cashmere goats, which are morphogenetically distinct from primary hair follicles (PHFs). Here, in light of having initially produced 15 T*β*4 overexpression (T*β*4-OE) cashmere goats which had more SHFs than the wild type (WT) goats, and produced more cashmere, we produced T*β*4-OE offsprings both via somatic cell nuclear transfer (SCNT) and via natural mating (NM). However, the desired trait exhibited lower fixation in the line-bred offspring compared to the SCNT offspring. Integrative analysis of methylation and transcriptional profiles showed that this might be due to the influence of methylation on the expression of differentially expressed genes (DEGs) between generations, which was mutually consistent with the results of the functional and pathway enrichment analysis of differentially methylated regions (DMRs) and DEGs. Overall, our study systematically describes the DNA methylation characteristics between generations of cashmere goats and provides a basis for improving genetic stability.

## 1. Introduction

Offspring, which can stably inherit the fine trait, always attract interests of researchers in line breeding throughout the world and great attentions of industry [1]. In recent years, with the development of molecular biology and gene editing technology, we can obtain a large number of individuals with excellent target traits [2,3,4,5,6]. Therefore, we are eager to pass this trait on to future generations, so as to expand its production scale and better serve human beings. However, due to the genetic stability of traits [7], it is not always possible to achieve the desired goals. In addition, the region and time of breed breeding also affect the final breeding process [8]. Therefore, we need to determine the reasons that affect the genetic stability of traits and effectively avoid genetic instability, so as to select and breed new strain lines with good production values for promotion and utilization.

DNA methylation alteration has been observed in various animal development stages and is considered to be a cause of genetic instability [9,10]. Global hypomethylation is frequently seen in highly and moderately repeated DNA sequences and plays a key role in chromosomal instability [7]. Hypermethylation in gene promoter regions, such as in exogenous genes, is usually related to gene silencing [11]. Therefore, studying the methylation degree of genes between generations can lay a foundation for determining the factors that affect genetic stability.

Cashmere is derived from the secondary hair follicles (SHFs) of cashmere goats, which are morphogenetically distinct from primary hair follicles (PHFs). In contrast to PHFs (which produce guard hairs), SHFs do not contain medulla and instead generate fibers that are soft and delicate [12,13]. Indeed, one of the key goals of cashmere goat breeding is to increase the number of SHFs carried by the goat.

Thymus beta 4 (T*β*4), first isolated by Goldstein and White from the thymus of a calf in 1966 [14] and consisting of 43 amino acid residues, is a highly conserved G-actin sequestering protein with a wide range of functions, such as promoting cell migration, angiogenesis, and wound healing [15]. Recent studies have shown that T*β*4 induces rapid hair growth around the wound margin and promotes hair growth on the dorsal skin of mice and on the depilated areas of rats [16,17]. Moreover, the overexpression of T*β*4 in hair follicles can improve cashmere yield in cashmere goats [18]. In the last 10 years, our team has initially produced 15 T*β*4 overexpression (T*β*4-OE) cashmere goats, which had more secondary hair follicles than the WT goats and produced more cashmere (unpublished observations).

In this study, the offspring of T*β*4-OE cashmere goats were bred through somatic cell nuclear transfer (SCNT) and natural mating (NM), and the genetic stability of their cashmere production performance was further evaluated. At the same time, the strategy of genome-wide methylation sequencing combined with transcriptome sequencing was adopted to analyze the methylation differences between the original generation and the offspring of the T*β*4-OE cashmere goats, thereby providing a theoretical basis for further explaining the genetic instability between generations of genetically modified cashmere goats.

## 2. Materials and Methods

### 2.1. Animals

Forty-two goats were used for this study, including three types T*β*4-OE parental generation (P0, a total of 2), T*β*4-OE first filial generation of 2014 (F1-1, a total of 11; via NM, F1-1-NM, a total of 9; via SCNT, F1-1-SCNT, a total of 2), and 2015 (F1-2, a total of 12; via NM, F1-2-NM, a total of 8; via SCNT, F1-2-SCNT, a total of 4), and WT first filial generation of 2014 (F1-1-WT, a total of 7) and 2015 (F1-2-WT, a total of 10) (Table 1). All goats were monitored for their body weights. Six typical representatives (F1-1-NM, F1-1-SCNT, F1-1-WT, F1-2-NM, F1-2-SCNT, F1-2-WT) were selected for the physiological indices and hair follicle tissue analysis. Two parents (P0-GM, a female and a male from P0) and their twin offspring (F1-GM, from F1-2-NM) were selected for an intergenerational methylation study. When the goats were two years old, we sampled them in September of that year. All experiments followed the National Research Council Guide for the Care and Use of Laboratory Animals (The third edition, promulgated on March 1, 2017). All protocols were approved by the Institutional Animal Care and Use Committee of Inner Mongolia University (Hohhot, China). All goats were kept at the Inner Mongolia YiWei White Cashmere Goat Limited Liability Company Breeding Farm at Ulan Town of Erdos in Inner Mongolia Autonomous Region, China.

### 2.2. Cell Culture

Goat fetal fibroblasts (GFbs) were isolated from 35-day-old fetal T*β*4-OE and WT goats as previously described [19]. The T*β*4-OE and WT GFbs, as the donor cells for SCNT, were cultured in Dulbecco’s modified Eagle’s medium/F12 containing 10% fetal bovine serum in 5% CO_2_ at 37 °C in a humidified incubator for 3–5 days and then passaged and frozen for future use.

### 2.3. Generation of Cloned Goats via Somatic Cell Nuclear Transfer (SCNT)

SCNT was performed as described previously [19]. Briefly, oocytes matured in vitro were enucleated, and selected polyclonal and donor cells were injected into the perivitelline space. The oocyte-donor cell pairs were fused and activated. After being cultured for 20 h, cloned embryos were surgically transferred into the oviducts of the surrogates.

### 2.4. Measurement of Body-Related Indices

The body mass of all first filial generation of T*β*4-OE and WT were determined at birth. Body mass, cashmere weight, cashmere thickness, and fiber length were monitored yearly until the goat died.

### 2.5. Blood Biochemistry

Subjugular venous blood was collected from six typical representatives in the morning after an overnight fast to minimize inter-individual variations due to metabolic effects. Heparin sodium salt-containing vacuum blood tubes (Kangjian Medical Apparatus Co. Ltd., Jiangsu Province, China) were used to prevent coagulation. Blood was centrifuged and examined for biochemical indices using a Glamour 3000 automatic biochemistry analyzer. Analytical measurements included routine blood count (white blood cell (WBC), red blood cells (RBC), hemoglobin (HGB) and, platelet (PLT)), liver function (gamma-glutamyltransferase (GGT), aspartate aminotransferase (AST), alanine transaminase (ALT), alkaline phosphatase (ALP), totaln protein (TPROT), albumin (ALB), globulin (GLOB), and total bilirubin (TBIL)) and renal function tests (blood urea nitrogen (BUN), serum creatinine (CRE), and blood uric acid (URIC)).

### 2.6. RNA Extraction and Quantitative Real Time-PCR (qRT-PCR)

Total RNA was isolated from tissue samples of six typical representatives, two parents and their twin offsprings with RNAiso Plus* (TaKaRa Bio, Shiga, Japan). cDNA was synthesized from 1 μg of total RNA with a PrimeScript RT reagent Kit with a gDNA Eraser (Perfect Real Time) (TaKaRa Bio, Shiga, Japan), following the manufacturer’s instructions. qPCR was performed using SYBR Premix Ex Taq II (TaKaRa Bio, Shiga, Japan) on a 7500 Real-Time PCR System (Applied Biosystems, Munich, Germany) [20].

### 2.7. Western Blot Analysis

Expression of the T*β*4 in the skin samples of six typical representatives was detected by western blot analysis. Total protein was extracted from the skin samples obtained from the body side of the goats, separated by 10% sodium dodecyl sulfate polyacrylamide gel electrophoresis, and transferred to a nitrocellulose membrane. This membrane was blocked with 5% skimmed milk at room temperature for 1 h and incubated overnight at 4 °C using an anti-T*β*4 antibody (1:1000 dilution) and an anti-GAPDH antibody (1:10,000 dilution) as a loading control. After incubation, the membrane was rinsed sequentially with phosphate-buffered saline and phosphate-buffered saline containing 0.05% Tween-20 solution. Subsequently, the membrane was treated with a secondary goat anti-rabbit antibody. Protein bands were visualized using a Tanon-5200 image-analysis system (Tanon Science and Technology Co., Ltd, Shanghai, China) [21].

### 2.8. Hematoxylin-Eosin (H&E) and Immunohistochemistry (IHC) Staining

Skin tissues obtained from the six typical representatives were prefixed with 4% paraformaldehyde for 48 h, dehydrated in a series of alcohol concentrations, transferred into xylene, and embedded in paraffin. We cut 5 mm sections from each embedded tissue samples, and stained the sections with H&E. Stained sections were dehydrated and sealed with a cover slip [2]. The T*β*4 expression levels were detected by immunohistochemistry following the previous description [22,23]. Moreover, the ratio of secondary and primary hair follicles (SHFs/PHFs ratio) in sections of skin tissue from six typical representatives was statistically determined.

### 2.9. DNA Extraction

Goat genomic DNA samples used for the whole-genome bisulphite sequencing (WGBS) were extracted and purified from skin tissues of two parents and their twin offsprings (200–500 mg per sample) using a DNA extraction kit (Promega, Madison, WS, USA). A total of 1 μg qualified gDNA (concentration ≥50 ng/μL, A260/A280 = 1.8–2.0, without RNA, protein and degradation) was used for each library construction. A K5500 spectrophotometer was used to detect the purity of DNA, and 1% agarose gel electrophoresis was used for genome integrity identification. A Qubit^®^ 3.0 Fluorometer was used for the quantitation of DNA.

### 2.10. Whole Genome Bisulphite Sequencing (WGBS) and Data Processing

The volume of the combined gDNA sample and unmethylated lambda DNA controlled was adjusted to a total volume of 80 μL using 1× TE, and the DNA was fragmented to around 300 bp. Blunt-ended fragments was created by filling in or chewing back 3’ and 5’ overhangs. Phosphorylation of the 5’ ends ensured that the fragments were suitable for ligation. The next step was dA-tailing of the 3’end of the end-repaired, phosphorylated fragments. The single A overhang enabled ligation to adaptors with single T overhangs. The methylated adaptor containing sequences required downstream in the sequencing workflow were ligated to the dA-tailed fragment. 2% agarose gel selected the 350–500 bp fragments. The bisulfite conversion technique involved treating DNA with bisulfite, which converted unmethylated cytosines into uracil. Methylated cytosines remained unchanged during the treatment. The uracil-binding pocket of KAPA HiFi DNA polymerase was inactivated, enabling amplification of uracil containing DNA. Furthermore, converted DNA fragments were PCR amplified and sequenced using HiSeq ×10 platform (Illumina, San Diego, CA, USA) [24]. To obtain high-quality clean reads, raw reads were filtered using the following criteria: (1) remove reads containing more than 10% unknown nucleotides (N); (2) remove low quality reads containing more than 40% low quality (Q-value ≤ 20) bases.

### 2.11. Methylation Level Analysis

The obtained clean reads were mapped to the species reference genome using the BSMAP software by default [25]. Then, a custom Perl script was used to determine the amounts of methylated cytosine and calculate methylation based on the methylated cytosine percentage in the entire genome, in each chromosome, and in different regions of the genome for each sequence’s context (CG, CHG, and CHH; H = A, C, or T). To assess the different methylation patterns in different genomic regions, methylation profiles at 5′-flanking 2kb regions and gene sequences (or transposable elements, TEs) were plotted based on the average methylation for each 100 bp interval.

### 2.12. Differentially Methylated Regions (DMRs) Analysis

The DMRs for each sequence context (CG, CHG, and CHH; H = A, C, or T) between the samples were identified according to the following stringent criteria: more than five methylated cytosine molecules in at least one sample; the total depth of sequencing for each methylation cytosine site was >10, and the depth of support for methylation cytosine was >4; the region length measured between 40 bp and 10 kb; the distance between the adjacent methylated sites was <200 bp; the fold change of the average amount of methylation was >2; the Pearson’s chi-square test (*χ*^2^ ) value was *p* ≤ 0.05. At the adjacent 2 kb (upstream or downstream) or body regions of the genes or TEs; the putative overlapping DMRs were sorted for further study.

### 2.13. Transcriptome Analysis

We generated sequencing reads of 12 mRNA libraries on a HiSeq X10 platform. After removing the reads, we retained >6 Gb clean data for each sample. The reads were aligned using Bowtie2 v2.2.3 based on the Capra_hircus_ARS1 reference genome [26]. We successfully aligned 88.89–98.13% of the paired reads with concordant alignments to the reference genome. The expected fragments per kilobase of transcript sequence per millions base pairs sequenced (FPKM) value for each gene was calculated to estimate expression, and DESeq2 v1.6.3 was used to identify the DEGs [27]. The obtained *p*-values were corrected using the Benjamini and Hochberg algorithm for controlling the false discovery rate; genes were considered differentially expressed when *q* ≤ 0.05 and |log2_ratio| ≥ 1.

### 2.14. Enrichment Analysis of Differentially Expressed Genes (DEGs) and Differentially Methylated Regions (DMRs)

We conducted Gene Ontology (GO) enrichment and Kyoto Encyclopedia of Genes and Genomes (KEGG) pathway analyses using the R package “clusterprofiler” [18]. GO terms or KEGG pathways with adjusted *p* < 0.05 were considered statistically significant.

### 2.15. Statistical Analyses

Means, SDs, and SEMs were analyzed using Graphpad Prism 7.0. Two-way analyses of variance (ANOVAs) with Dunnett’s multiple comparison tests were used to compare the statistical differences in groups. We considered *p*-values <0.05 statistically significant.

## 3. Results

### 3.1. Establishment of the Germline of Thymosin β4 Overexpression (Tβ4-OE) Cashmere Goats

**Figure 1:**
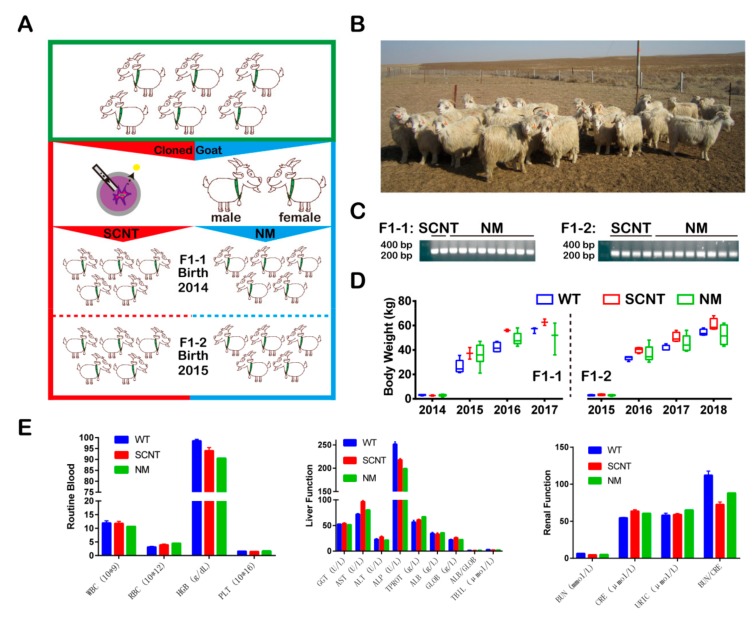
Establishment of the thymosin *β*4 overexpression (T*β*4-OE) germline. (**A**) A schematic showing germline transmission. (**B**) T*β*4-OE first filial generation of 2014 (F1-1, a total of 11; via NM, F1-1-NM, a total of 9; via SCNT, F1-1-SCNT, a total of 2), and 2015 (F1-2, a total of 12; via NM, F1-2-NM, a total of 8; via SCNT, F1-2-SCNT, a total of 8). (**C**) PCR genotyping, showing the insertion of donor DNA in the T*β*4-OE goats. SCNT: offspring produced by somatic nuclear transfer: NM: offsprings produced by natural mating. (**D**) Body weights of the F1-1 and F1-2 from birth to four years old. CON: control. (**E**) Physiological indexes of the F1-1 and F1-2. Analytical measurements included routine blood count (white blood cell (WBC), red blood cells (RBC), hemoglobin (HGB) and, platelet (PLT)), liver function (gamma-glutamyltransferase (GGT), aspartate aminotransferase (AST), alanine transaminase (ALT), alkaline phosphatase (ALP), total protein (TPROT), albumin (ALB), globulin (GLOB), and total bilirubin (TBIL)) and renal function tests (blood urea nitrogen (BUN), serum creatinine (CRE), and blood uric acid (URIC).

To investigate whether the desired trait (i.e., increased cashmere yield) could be stably inherited, we produced T*β*4-OE offspring both via SCNT and via NM (Figure 1A). Using these methods, we successfully established a flock of 23 goats, all of which overexpressed T*β*4 (Figure 1B,C). Of these, there were 11 F1-1 (two via SCNT and nine via NM) and 12 F1-2 (4 via SCNT and 8 via NM) (Table 2 and Table 3). We monitored the body weights of all T*β*4-OE cashmere goats and physiological indices of the six typical representatives; the average body weights and physiological indices of the F1-T*β*4-OE were not significantly different from those of the F1-WT goats of the same age (*p* > 0.05; Figure 1D,E). This suggests that the T*β*4-OE offspring are just as healthy as the WT ones.

### 3.2. Cashmere Yield and SHFs/PHFs Ratio Increased in F1-SCNT

**Figure 2:**
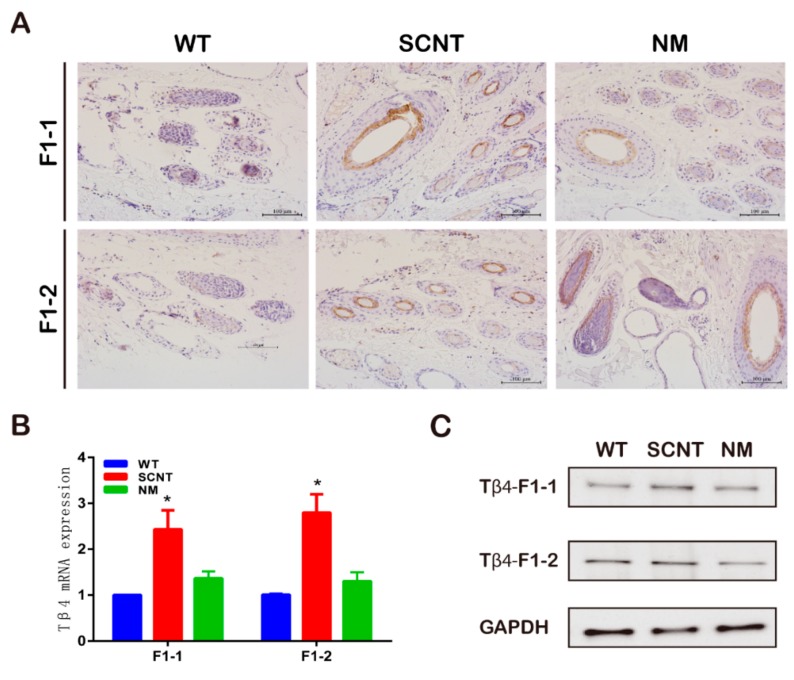
Comparison of T*β*4 expression in the hair follicles of T*β*4-OE first filial generation. (**A**) Immunohistochemical (IHC) staining of T*β*4 expression in representative samples of hair follicles; hair follicles of F1-1 and F1-2 cashmere goats are shown; F1-1, T*β*4-OE first filial generation of 2014; F1-2, T*β*4-OE first filial generation of 2015 (**B**) qPCR and (**C**) western blot showing T*β*4 expression in the skin of F1-1 and F1-2 cashmere goats. Asterisks indicate that the mean expression was significantly different from the control (*, *p* < 0.05). Bars indicate the means of three replicates; error bars indicate the standard error of the mean.

To investigate whether T*β*4 is expressed in the hair follicles, we used IHC to detect T*β*4 expression in hair follicles. The results suggested greater T*β*4 expression in the hair follicles of the F1-SCNT compared to the WT and F1-NM (Figure 2A). Meanwhile, our qPCR and western blot analyses also indicated that the T*β*4 protein and mRNA expression levels were higher in the F1-SCNT goats than in the F1-WT and F1-NM goats (Figure 2B).

**Figure 3:**
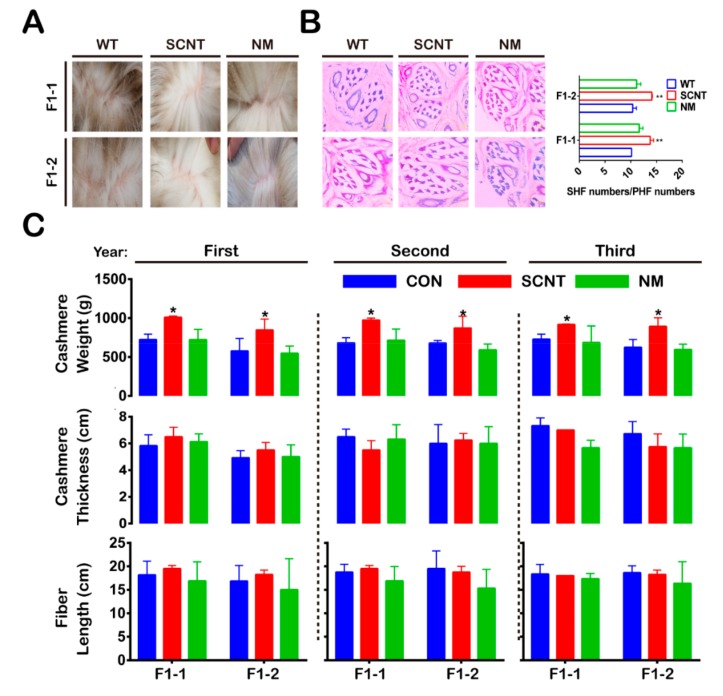
Comparison of the number of hair follicles and cashmere indicators of T*β*4-OE first filial generation. (**A**) Comparison of the cashmere growth in different offspring produced by SCNT and NM; SCNT, somatic cell nuclear transfer; NM, natural mating; WT, wild type. (**B**) Hematoxylin-eosin (H&E) staining of the backside skin from F1-1 and F1-2 cashmere goats; F1-1, T*β*4-OE first filial generation of 2014; F1-2, T*β*4-OE first filial generation of 2015; PHF, primary hair follicle; SHF, secondary hair follicles (**, *p* < 0.01). (**C**) Cashmere weight, cashmere thickness, and fiber length for the F1-1 and F1-2 goats; CON, control (*, *p* < 0.05).

Moreover, we investigated whether F1-T*β*4-OE had more hair follicles than the F1-WT. We found that the SHFs/PHFs ratio in the F1-SCNT was significantly greater than that of the F1-NM and the F1-WT (Figure 3A,B). We also measured the cashmere’s weight, thickness, and fiber length for all F1-T*β*4-OE and F1-WT goats (Figure 3C). The average cashmere yield of the F1-SCNT was significantly greater than that of the F1-NM, as well as that of the F1-WT (*p* < 0.05). There were no significant differences in the cashmere thickness and fiber length among the three groups of goats.

### 3.3. Differential Analysis of Methylation and Expression

**Figure 4:**
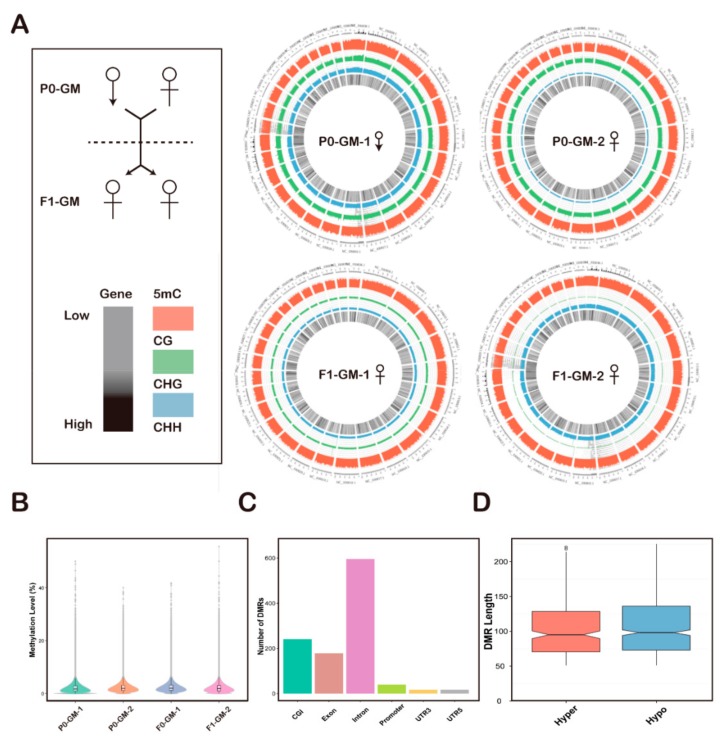
Methylation level profiles for the skin samples of cashmere goats. (**A**) Circus plot showing the genome-wide relationship between the DNA methylation and gene expression of the skin samples. The outermost ring displays the chromosome numbers and bands. The second ring (red) shows the methylation of CHG (H = A, C, or T). The third ring (green) shows the methylation of CG. The fourth ring (blue) shows the methylation of CHH (H = A, C, or T). The inner most circle (black) represents gene expression. 5mC, 5-methylcytosine. P0-GM-1, a male from P0 for an generational methylation study; P0-GM-2, a female from P0 for an generational methylation study; F1-GM-1, the one of twin offspring from F1-2-NM for an generational methylation study; F1-GM-2, the another of twin offspring from F1-2-NM for an generational methylation study. (**B**) a violin plot showing the methylation levels of the skin samples with 2kb sliding windows. (**C**) Distribution of differentially methylated CpGs and genes of differentially methylated regions (DMRs); CGI, CpG iland. (**D**) Comparison of DMR length distribution between hyper and hypo. Hyper, hypermethylation; Hypo, hypomethylation.

In order to investigate the genetic stability of the cashmere traits between generations of cashmere goats, we selected the two parents (P0-GM, a female and a male from P0 for an generational methylation study) and their twin offspring (F1-GM, from F1-2-NM for an generational methylation study) as research models. Methylation data of the P0-GM and F1-GM skin samples from *WGBS* were used for differential methylation analysis. There were 336 hypermethylated and 753 hypomethylated mC between the P0-GM and F1-GM, which correspond to 214 hypermethylated and 560 hypomethylated genes. Subsequently, the distribution of the methylation level of methylated C base was described based on the chromosome level, and a Circos diagram was drawn (Figure 4A). Interestingly, we observed that the F1-GM had thinner CHG and CHH than the P0-GM. Then, to further understand the distribution of the methylation level in the whole genome, the methylation level in the 2Kb window of the whole gene was calculated, and a Violin graph was drawn (Figure 4B). We analyzed DMRs distribution in different genomic regions (Figure 4C). The largest numbers were on the introns. Meanwhile, the hyper and hypo DMRs lengths were calculated (Figure 4D).

**Figure 5:**
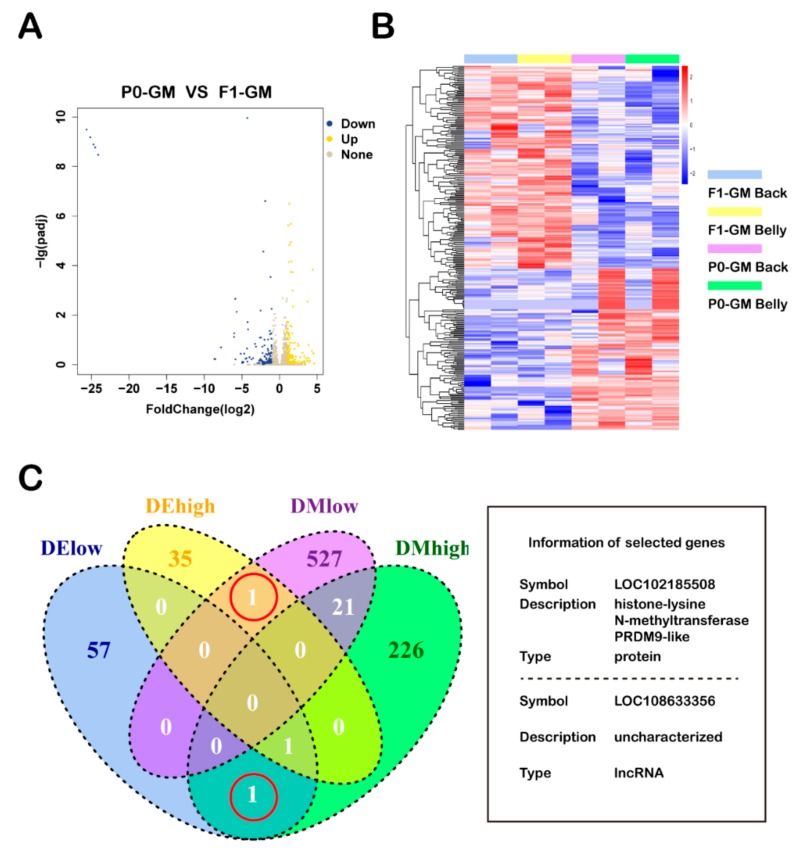
Transcriptional level profiles for the skin samples of cashmere goats and an integrative analysis of the methylation and transcriptional profiles. (**A**) A volcano plot of all genes in the skin samples from the P0-GM and F1-GM cashmere goats; showing genes with >2-fold difference and an adjusted *p* < 0.01 among groups; P0-GM, the two parents (a female and a male from P0 for an generational methylation study); F1-GM, their twin offspring (from F1-2-NM for an generational methylation study). (**B**) The heat map of gene expression profiles in the skin samples from the P0-GM and F1-GM cashmere goats. The colored bars illustrate relative expression. In the map, each sample group is clustered. Skin samples were taken from the back and belly of cashmere goats. (**C**) A Venn diagram illustrating a comparison of differentially methylated genes and differentially expressed genes. The red circles represent the genes selected. The box shows information about the genes selected. DE-high, the differentially expressed genes (DEGs) of high expressed; DE-low, the DEGs of low expressed; DM-high, differentially methylated regions (DMRs) of hypermethylation; DM-low, DMRs of hypomethylation.

Expression data for the P0-GM and F1-GM skin samples from sequencing were used for differential expression analysis. We found 36 highly expressed (‘DE-high’) and 58 lowly expressed (‘DE-low’) genes in F1-GM skin samples (Figure 5A,B).

### 3.4. Roles of Methylation in Regulating Gene Expression

We analyzed the intersection between differentially expressed genes and differentially methylated genes. One gene was hypermethylated with a low expression in HCC (NCBI query: Symbol, LOC102185508; Description, histone-lysine N-methyltransferase PRDM9-like; Type, protein), and one gene was hypomethylated with high expression (NCBI query: Symbol, LOC108633356; Description, uncharacterized; Type, lncRNA) (Figure 5C).

### 3.5. Comparison of Functional Analysis of DEGs and DMRs with GO and KEGG

**Figure 6:**
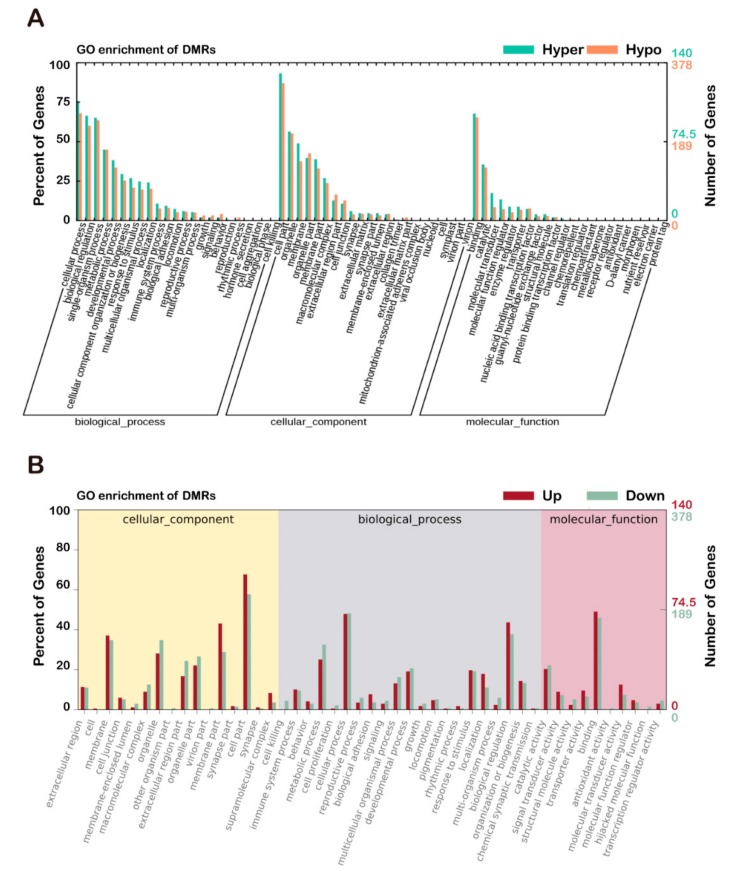
Comparison of the GO analysis of DMRs and DEGs. (**A**) GO analysis of DMRs. (**B**) GO analysis of DEGs. GO, Gene ontology enrichment analysis; DMRs, differentially methylated regions; DEGs, differentially expressed genes.

A functional enrichment analysis of genes with overlapping promoter and genomic region with DMRs was carried out by R. The GO analysis results showed that changes in the biological processes (BP) of DMRs were significantly enriched in the cellular process, biological regulation, and metabolic process. Changes in the molecular function (MF) were mainly enriched in binding, catalytic activity, and molecular transducer activity. Changes in the cell component (CC) of DMRs were mainly enriched in the cell part, organelle, and membrane (Figure 6A). Interestingly, the above analysis is highly consistent with the results of the GO function enrichment of DEGs in the P0-GM and F1-GM cashmere goats (Figure 6B).

**Figure 7:**
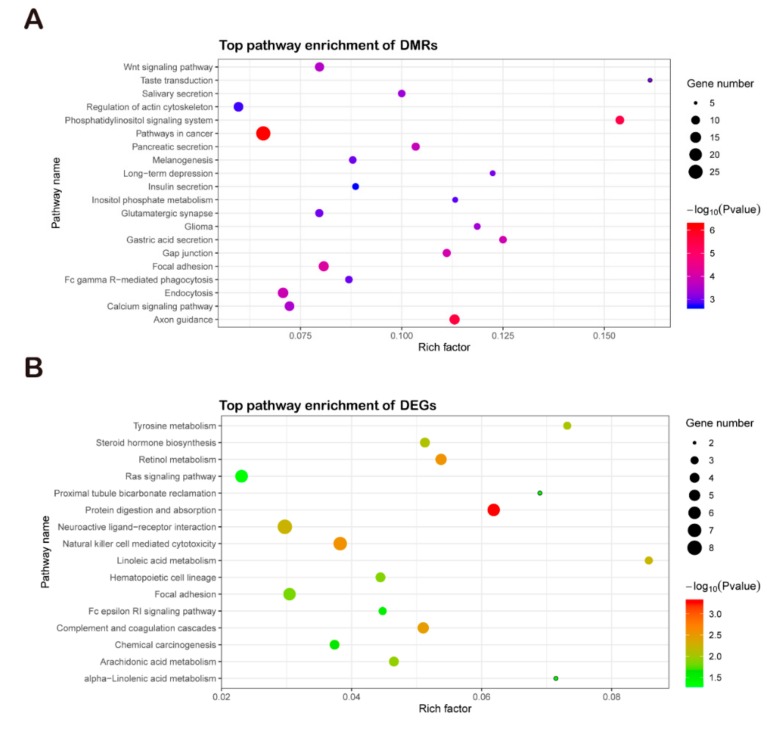
Comparison of KEGG analysis of DMRs and DEGs. (**A**) KEGG analysis of DMRs. (**B**) KEGG analysis of DEGs. KEGG, Encyclopedia of Genes and Genomes enrichment analysis; DMRs, differentially methylated regions; DEGs, differentially expressed genes.

A KEGG pathway analysis revealed that the DMRs were mainly enriched in pathways in cancer, axon guidance, and the phosphatidylinositol signaling system (Figure 7A). Meanwhile, the KEGG pathway analysis revealed that the DEGs were mainly enriched in protein digestion and absorption, complement and coagulation cascades, and natural killer cell mediated cytotoxicity (Figure 7B).

## 4. Discussion

For the past 10 years, we have studied the effects of T*β*4 gene overexpression on cashmere goat hair follicles, in order to select and breed high-yield cashmere goats. After observing that T*β*4 overexpression had a significant effect on cashmere yield in cashmere goats [18], we used SCNT and natural breeding methods to develop a new goat breed that stably inherited overexpression of T*β*4. This was particularly difficult because, in addition to the many general constraints on large-scale mammal breeding (such as the environment and the operation), unfavorable factors are particularly prominent on the Inner Mongolia Plateau, which has a fragile ecological environment, drastically fluctuating temperatures, sparse vegetation, harsh winds, and sandy soils [28]. Despite these challenges, we have to date successfully established a flock of nearly 40 T*β*4-OE cashmere goats. Importantly, the T*β*4-OE goats were healthy, with normal growth rates, normal activity levels, and no obvious behavioral defects. This is related to the insertion of a promoter (KAP6.1) specific to the expression of T*β*4 in the hair follicle into the vector. Therefore, it did not affect the embryonic development of cashmere goats. Thus, this flock provides a solid foundation for further analysis of the genetic stability of the T*β*4 gene over several generations.

We carefully selected the six representatives for study based on the properties of cashmere among first filial generation of T*β*4-OE and WT goats. Each representative fully reflects the average level of trait of that type. Therefore, the differences among different types are reflected in the results. Meanwhile, the goats for the intergenerational methylation study were rare and unique. We selected two T*β*4 overexpressed cashmere goats (a male and a female) for mating and obtained twin offspring. Such groupings are difficult to obtain, since cashmere goats have little chance of producing double lambs, especially genetically modified ones, and such groupings can eliminate interference, especially the temporal and spatial interference between individuals.

The advantage of SCNT is that this procedure transfers the parental genome to the offspring without alteration; its disadvantage remains its low production efficiency [29,30,31]. This was reflected by the results of our offspring breeding program: the SCNT T*β*4-OE offspring were equivalent to their parents in all measured traits, but only a small number of these offspring were successfully born. In contrast, a large number of NM T*β*4-OE offspring were successfully born. However, NM T*β*4-OE offspring lost some of the desired traits. Our study confirmed that the genetic instability between generations was caused by methylation regulating the expression of some genes, such as the LOC102185508 and LOC108633356 obtained by the combined analysis of transcriptome and methylation. Although these effects may lead to genetic instability, NM also produced far more live offspring than SCNT. Therefore, it is most effective to improve livestock genomes by combining SCNT and NM.

In addition, we found that the GO function enrichment results of DMRs and DEGs were highly similar. This suggests that intergenerational methylation changes affect the genetic stability of traits by regulating intergenerational DEGs. This suggests that we can improve the genetic stability of traits by changing the methylation degree of DEGs between generations. This result still needs further experimental verification. However, the inconsistency of the KEGG results also indicates that DMRs and DEGs use different paths to affect the genetic stability of traits.

## 5. Conclusions

In summary, the desired trait exhibited lower fixation in the line-bred offspring compared to the SCNT offspring. An integrative analysis of the methylation and transcriptional profiles showed that this result might be due to the influence of methylation on the expression of DEGs between generations, which was mutually consistent with the results of the GO and KEGG analysis of DMRs and DEGs. To eliminate the effects of DNA methylation, it would be more efficient to produce animals carrying a gene integrated at specific genomic loci. It is clear CRISPR/Cas9-based genome engineering will accelerate improvements in desirable livestock traits. The use of CRISPR/Cas9 avoids the target gene silencing caused by methylation and will ensure the stable expression of the target gene and the presence of the target trait in all offspring.

## Figures and Tables

**Table 1:** Goats used for this study

Birth Year	Type	Group	Number	Number of Experiments-1 ^12^	Number of Experiments-2 ^13^	Number of Experiments-3 ^14^
2010	P0 ^1^	P0-GM ^4^	2	NA ^15^	NA ^15^	2
2014	F1-1 ^2^	F1-1-NM ^5^	9	9	1	NA ^15^
		F1-1-SCNT ^6^	2	2	1	NA ^15^
		F1-1-WT ^7^	7	7	1	NA ^15^
2015	F1-2 ^3^	F1-2-NM ^8^/F1-GM ^11^	8	8	1	2
		F1-2-SCNT ^9^	4	4	1	NA ^15^
		F1-2-WT ^10^	10	10	1	NA ^15^
Overall			42	40	7	4

^1^ P0, T*β*4-OE parental generation; ^2^ F1-1, T*β*4-OE first filial generation of 2014; ^3^ F1-1, T*β*4-OE first filial generation of 2015; ^4^ P0-GM, a female and a male from P0 for a generational methylation study; ^5^ F1-1-NM, T*β*4-OE first filial generation of 2014 via natural mating (NM); ^6^ F1-1-SCNT, T*β*4-OE first filial generation of 2014 via somatic cell nuclear transfer (SCNT); ^7^ F1-1-WT, wild type (WT) first filial generation of 2014; ^8^ F1-2-NM, T*β*4-OE first filial generation of 2015 via NM; ^9^ F1-2-SCNT, T*β*4-OE first filial generation of 2015 via SCNT; ^10^ F1-2-WT, WT first filial generation of 2015; ^11^ F1-GM, from F1-2-NM for a generational methylation study; ^12^ experiments-1, the experiments for measurement of body-related indices; ^13^ experiments-2, the experiments for the physiological indices and hair follicle tissue analysis; ^14^ experiments-3, the experiments for an intergenerational methylation study; ^15^ NA, not available.

**Table 2:** F1-1 and F1-2 SCNT embryonic statistics.

Group	Mature ^2^			Clone ^3^			Fusion ^4^			Cleavage ^5^					
	Failure (n)	Success (n)	Ratio (%)	Failure (n)	Success (n)	Ratio (%)	Failure (n)	Success (n)	Ratio (%)	Failure (n)	2C ^6^ (n)	4C ^7^ (n)	≥8C ^8^ (n)	Success (n)	Ratio (%)
2014 SCNT ^1^	86	150	63.56	19	131	87.33	33	98	74.81	44	0	47	7	54	55.10
	65	118	64.48	13	105	88.98	26	79	75.24	18	0	42	19	61	77.22
	50	85	62.96	17	68	80.00	18	50	73.53	29	0	12	9	21	42.00
	41	82	66.67	4	78	95.12	19	59	75.64	25	0	14	20	34	57.63
	40	56	58.33	2	54	96.43	15	39	72.22	19	0	6	14	20	51.28
	30	35	53.85	2	33	94.29	8	25	75.76	13	0	4	8	12	48.00
	50	68	57.63	15	53	77.94	20	33	62.26	23	0	9	1	10	30.30
	32	117	78.52	17	100	85.47	30	70	70.00	42	0	20	8	28	40.00
	46	70	60.34	27	43	61.43	6	37	86.05	23	0	8	6	14	37.84
	28	42	60.00	1	41	97.62	18	23	56.10	15	0	8	0	8	34.78
	16	49	75.38	7	42	85.71	13	29	69.05	14	0	13	2	15	51.72
	53	39	42.39	7	32	82.05	14	18	56.25	5	0	9	4	13	72.22
**Overall**	**537**	**911**	**62.91**	**131**	**780**	**85.62**	**220**	**560**	**71.79**	**270**	**0**	**192**	**98**	**290**	**51.79**
2015 SCNT ^1^	27	79	74.53	0	79	100.00	27	52	65.82	40	12	0	0	12	23.08
	54	130	70.65	0	130	100.00	55	75	57.69	66	9	0	0	9	12.00
	46	119	72.12	0	119	100.00	42	77	64.71	35	42	0	0	42	54.55
	51	93	64.58	0	93	100.00	40	53	56.99	43	4	0	0	4	8.51
	36	107	74.83	0	107	100.00	49	58	54.21	41	17	0	0	17	29.31
	28	135	82.82	0	135	100.00	67	68	50.37	30	32	0	0	32	51.61
	33	118	78.15	0	118	100.00	45	73	61.86	47	23	0	0	23	32.86
	36	84	70.00	0	84	100.00	21	63	75.00	37	23	0	0	23	38.33
**Overall**	**311**	**865**	**73.55**	**0**	**865**	**100.00**	**346**	**519**	**60.00**	**339**	**162**	**0**	**0**	**162**	**32.34**

^1^ SCNT, somatic cell nuclear transfer; ^2^ Mature, oocytes that were cultured to mature in vitro; ^3^ Clone, oocytes that transferred somatic cell nuclear; ^4^ Fusion, embryos that were fused; ^5^ Cleavage, cleavage embryos; ^6^ 2C, two-cell stage; ^7^ 4C, four-cell stage; ^8^≥8C, after eight-cell stage.

**Table 3:** Goat birth statistics

Birth Year	Group	Oocytes(n)	SCNT ^1^ Embryos (n)	Embryos Transferred (n)	Surrogates (n)	Pregnancies (n)	Live Births (n)	Surviving Offspring (n)	Sex (♀/♂)
2014	F1-1-NM ^2^	NA ^8^	NA ^8^	NA ^8^	NA ^8^	7	9	9	1/8
	F1-1-SCNT ^3^	1448	290	177	46	2	2	2	0/2
	F1-1-1-WT ^4^	NA ^8^	NA ^8^	NA ^8^	NA ^8^	4	7	7	1/6
2015	F1-2-NM ^5^	NA ^8^	NA ^8^	NA ^8^	NA ^8^	5	8	8	5/3
	F1-2-SCNT ^6^	1176	501	367	99	4	4	4	2/2
	F1-2-WT ^7^	NA ^8^	NA ^8^	NA ^8^	NA ^8^	6	10	10	3/7

^1^ SCNT, somatic cell nuclear transfer; ^2^ F1-1-NM, T*β*4-OE first filial generation of 2014 via natural mating (NM); ^3^ F1-1-SCNT, T*β*4-OE first filial generation of 2014 via somatic cell nuclear transfer (SCNT); ^4^ F1-1-WT, wild type (WT) first filial generation of 2014; ^5^ F1-2-NM, T*β*4-OE first filial generation of 2015 via NM; ^6^ F1-2-SCNT, T*β*4-OE first filial generation of 2015 via SCNT; ^7^ F1-2-WT, WT first filial generation of 2015; ^8^ NA: not available.
